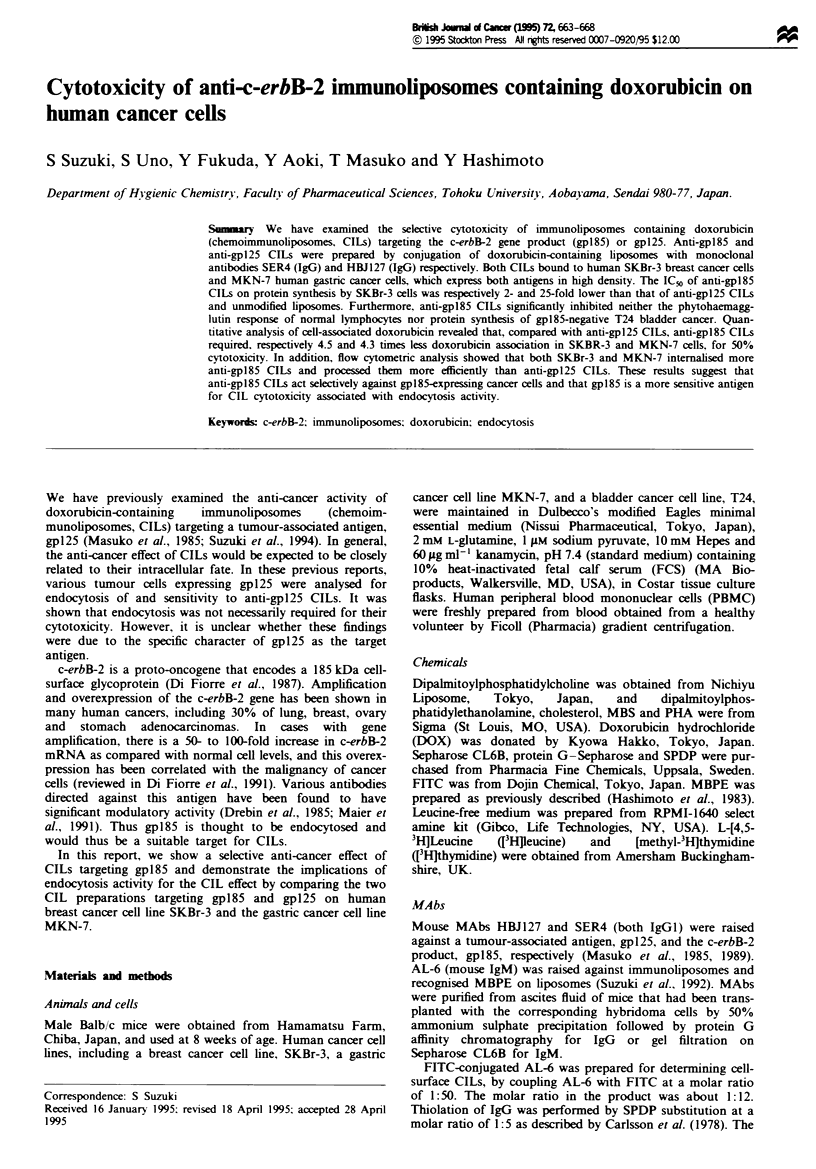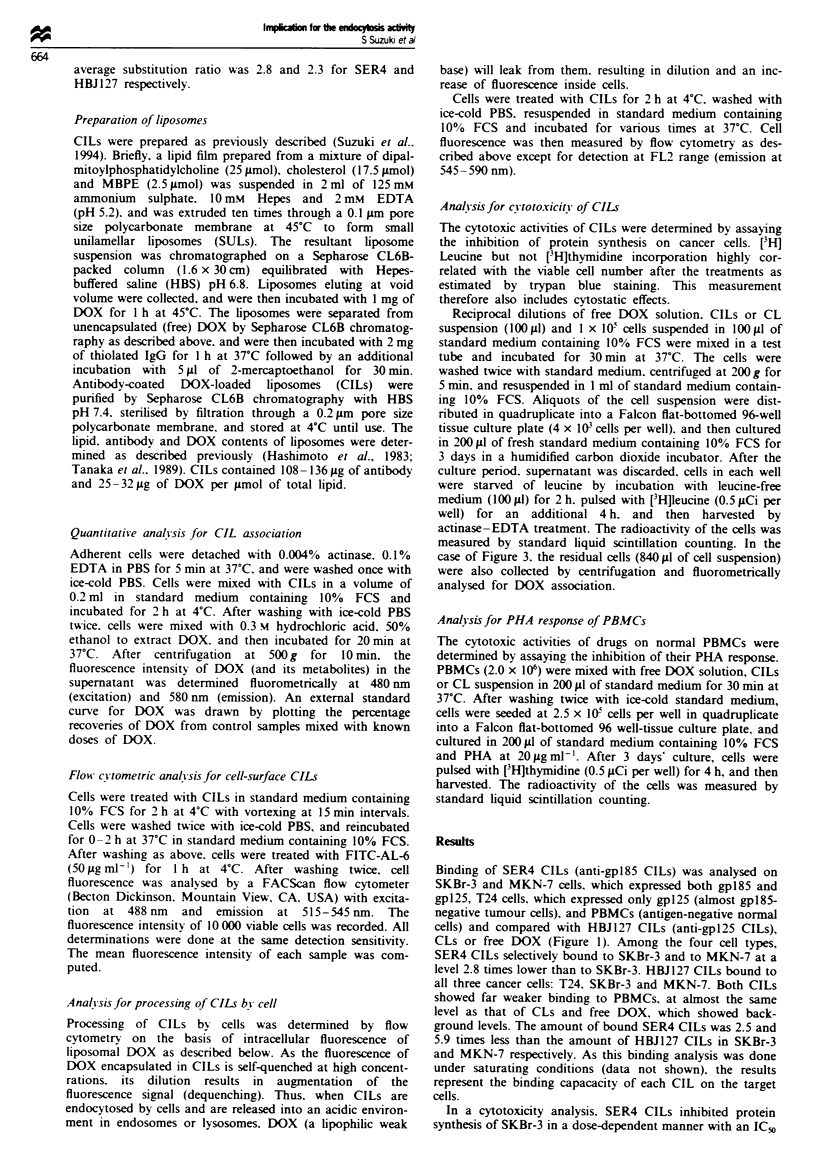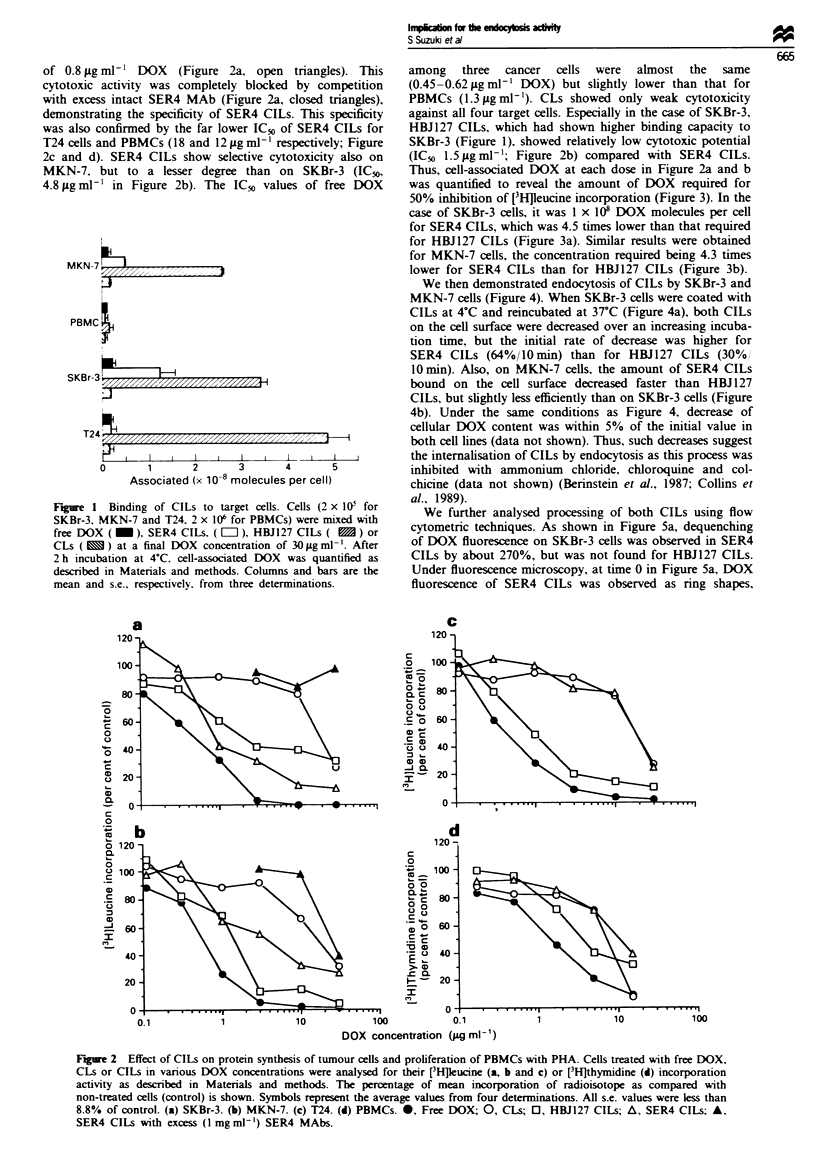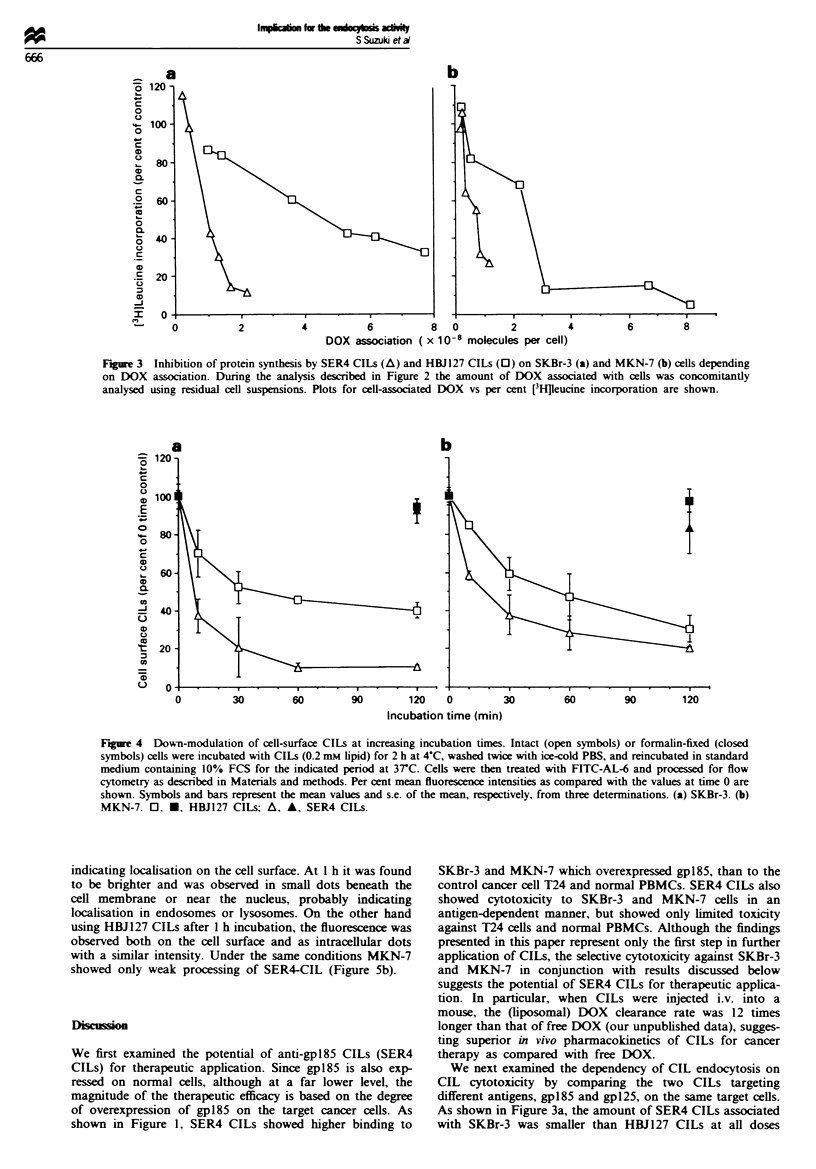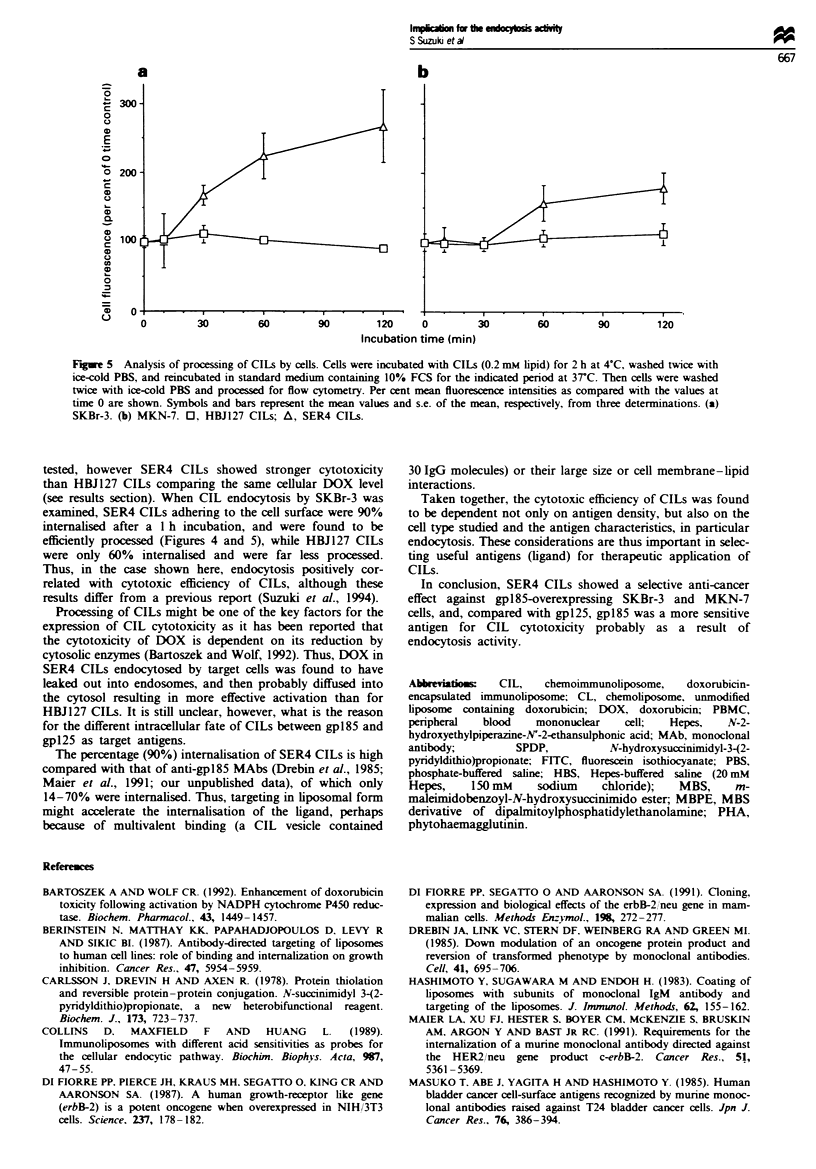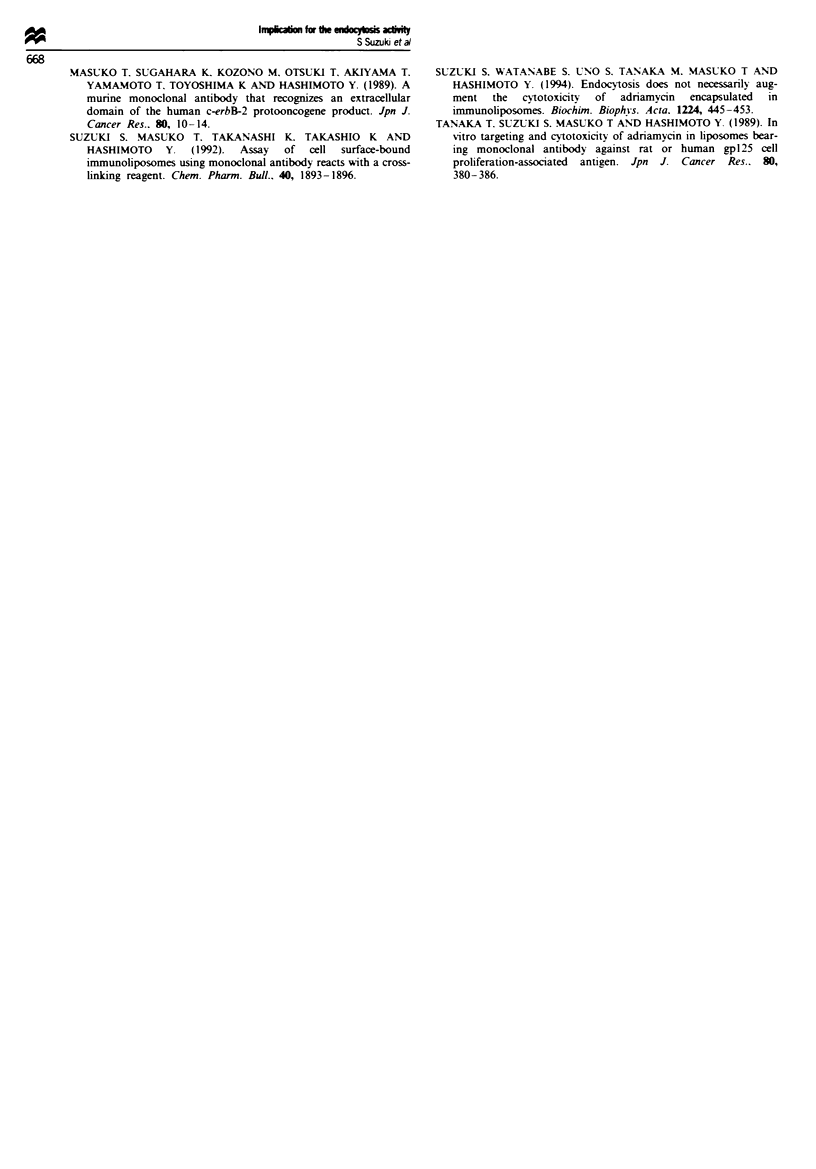# Cytotoxicity of anti-c-erbB-2 immunoliposomes containing doxorubicin on human cancer cells.

**DOI:** 10.1038/bjc.1995.391

**Published:** 1995-09

**Authors:** S. Suzuki, S. Uno, Y. Fukuda, Y. Aoki, T. Masuko, Y. Hashimoto

**Affiliations:** Department of Hygienic Chemistry, Faculty of Pharmaceutical Sciences, Tohoku University, Sendai, Japan.

## Abstract

We have examined the selective cytotoxicity of immunoliposomes containing doxorubicin (chemoimmunoliposomes, CILs) targeting the c-erbB-2 gene product (gp185) or gp125. Anti-gp185 and anti-gp125 CILs were prepared by conjugation of doxorubicin-containing liposomes with monoclonal antibodies SER4 (IgG) and HBJ127 (IgG) respectively. Both CILs bound to human SKBr-3 breast cancer cells and MKN-7 human gastric cancer cells, which express both antigens in high density. The IC50 of anti-gp185 CILs on protein synthesis by SKBr-3 cells was respectively 2- and 25-fold lower than that of anti-gp125 CILs and unmodified liposomes. Furthermore, anti-gp185 CILs significantly inhibited neither the phytohaemagglutin response of normal lymphocytes nor protein synthesis of gp185-negative T24 bladder cancer. Quantitative analysis of cell-associated doxorubicin revealed that, compared with anti-gp125 CILs, anti-gp185 CILs required, respectively 4.5 and 4.3 times less doxorubicin association in SKBR-3 and MKN-7 cells, for 50% cytotoxicity. In addition, flow cytometric analysis showed that both SKBr-3 and MKN-7 internalised more anti-gp185 CILs and processed them more efficiently than anti-gp125 CILs. These results suggest that anti-gp185 CILs act selectively against gp185-expressing cancer cells and that gp185 is a more sensitive antigen for CIL cytotoxicity associated with endocytosis activity.


British Journal of Cancer (1995) 72, 663-668

© 1995 Stockton Press All rights reserved 0007-0920/95 $12.00

### Cytotoxicity of anti-c-*erb*B-2 immunoliposomes containing doxorubicin on human cancer cells

S Suzuki, S Uno, Y Fukuda, Y Aoki, T Masuko and Y Hashimoto

*Department of Hygienic Chemistry, Faculty of Pharmaceutical Sciences, Tohoku University, Aobayama, Sendai 980-77, Japan.*

**Summary** We have examined the selective cytotoxicity of immunoliposomes containing doxorubicin (chemoimmunoliposomes, CILs) targeting the c-*erb*B-2 gene product (gp185) or gp125. Anti-gp185 and anti-gp125 CILs were prepared by conjugation of doxorubicin-containing liposomes with monoclonal antibodies SER4 (IgG) and HBJ127 (IgG) respectively. Both CILs bound to human SKBr-3 breast cancer cells and MKN-7 human gastric cancer cells, which express both antigens in high density. The $IC_{50}$ of anti-gp185 CILs on protein synthesis by SKBr-3 cells was respectively 2- and 25-fold lower than that of anti-gp125 CILs and unmodified liposomes. Furthermore, anti-gp185 CILs significantly inhibited neither the phytohaemagglutin response of normal lymphocytes nor protein synthesis of gp185-negative T24 bladder cancer. Quantitative analysis of cell-associated doxorubicin revealed that, compared with anti-gp125 CILs, anti-gp185 CILs required, respectively 4.5 and 4.3 times less doxorubicin association in SKBR-3 and MKN-7 cells, for 50% cytotoxicity. In addition, flow cytometric analysis showed that both SKBr-3 and MKN-7 internalised more anti-gp185 CILs and processed them more efficiently than anti-gp125 CILs. These results suggest that anti-gp185 CILs act selectively against gp185-expressing cancer cells and that gp185 is a more sensitive antigen for CIL cytotoxicity associated with endocytosis activity.

**Keywords:** c-*erb*B-2; immunoliposomes; doxorubicin; endocytosis

We have previously examined the anti-cancer activity of doxorubicin-containing immunoliposomes (chemoimmunoliposomes, CILs) targeting a tumour-associated antigen, gp125 (Masuko *et al.*, 1985; Suzuki *et al.*, 1994). In general, the anti-cancer effect of CILs would be expected to be closely related to their intracellular fate. In these previous reports, various tumour cells expressing gp125 were analysed for endocytosis of and sensitivity to anti-gp125 CILs. It was shown that endocytosis was not necessarily required for their cytotoxicity. However, it is unclear whether these findings were due to the specific character of gp125 as the target antigen.

c-*erb*B-2 is a proto-oncogene that encodes a 185 kDa cell-surface glycoprotein (Di Fiorre *et al.*, 1987). Amplification and overexpression of the c-*erb*B-2 gene has been shown in many human cancers, including 30% of lung, breast, ovary and stomach adenocarcinomas. In cases with gene amplification, there is a 50- to 100-fold increase in c-*erb*B-2 mRNA as compared with normal cell levels, and this overexpression has been correlated with the malignancy of cancer cells (reviewed in Di Fiorre *et al.*, 1991). Various antibodies directed against this antigen have been found to have significant modulatory activity (Drebin *et al.*, 1985; Maier *et al.*, 1991). Thus gp185 is thought to be endocytosed and would thus be a suitable target for CILs.

In this report, we show a selective anti-cancer effect of CILs targeting gp185 and demonstrate the implications of endocytosis activity for the CIL effect by comparing the two CIL preparations targeting gp185 and gp125 on human breast cancer cell line SKBr-3 and the gastric cancer cell line MKN-7.

### Materials and methods

### *Animals and cells*

Male Balb/c mice were obtained from Hamamatsu Farm, Chiba, Japan, and used at 8 weeks of age. Human cancer cell lines, including a breast cancer cell line, SKBr-3, a gastric cancer cell line MKN-7, and a bladder cancer cell line, T24, were maintained in Dulbecco's modified Eagles minimal essential medium (Nissui Pharmaceutical, Tokyo, Japan), 2 mM L-glutamine, 1 μM sodium pyruvate, 10 mM Hepes and 60 μg $ml^{-1}$ kanamycin, pH 7.4 (standard medium) containing 10% heat-inactivated fetal calf serum (FCS) (MA Bioproducts, Walkersville, MD, USA), in Costar tissue culture flasks. Human peripheral blood mononuclear cells (PBMC) were freshly prepared from blood obtained from a healthy volunteer by Ficoll (Pharmacia) gradient centrifugation.

### *Chemicals*

Dipalmitoylphosphatidylcholine was obtained from Nichiyu Liposome, Tokyo, Japan, and dipalmitoylphosphatidylethanolamine, cholesterol, MBS and PHA were from Sigma (St Louis, MO, USA). Doxorubicin hydrochloride (DOX) was donated by Kyowa Hakko, Tokyo, Japan. Sepharose CL6B, protein G-Sepharose and SPDP were purchased from Pharmacia Fine Chemicals, Uppsala, Sweden. FITC was from Dojin Chemical, Tokyo, Japan. MBPE was prepared as previously described (Hashimoto *et al.*, 1983). Leucine-free medium was prepared from RPMI-1640 select amine kit (Gibco, Life Technologies, NY, USA). L-[4,5-$^3$H]Leucine ([$^3$H]leucine) and [methyl-$^3$H]thymidine ([$^3$H]thymidine) were obtained from Amersham Buckinghamshire, UK.

### *MAbs*

Mouse MAbs HBJ127 and SER4 (both IgG1) were raised against a tumour-associated antigen, gp125, and the c-*erb*B-2 product, gp185, respectively (Masuko *et al.*, 1985, 1989). AL-6 (mouse IgM) was raised against immunoliposomes and recognised MBPE on liposomes (Suzuki *et al.*, 1992). MAbs were purified from ascites fluid of mice that had been transplanted with the corresponding hybridoma cells by 50% ammonium sulphate precipitation followed by protein G affinity chromatography for IgG or gel filtration on Sepharose CL6B for IgM.

FITC-conjugated AL-6 was prepared for determining cell-surface CILs, by coupling AL-6 with FITC at a molar ratio of 1:50. The molar ratio in the product was about 1:12. Thiolation of IgG was performed by SPDP substitution at a molar ratio of 1:5 as described by Carlsson *et al.* (1978). The

Correspondence: S Suzuki
Received 16 January 1995; revised 18 April 1995; accepted 28 April 1995

average substitution ratio was 2.8 and 2.3 for SER4 and HBJ127 respectively.

*Preparation of liposomes*

CILs were prepared as previously described (Suzuki *et al.*, 1994). Briefly, a lipid film prepared from a mixture of dipalmitoylphosphatidylcholine (25 μmol), cholesterol (17.5 μmol) and MBPE (2.5 μmol) was suspended in 2 ml of 125 mM ammonium sulphate, 10 mM Hepes and 2 mM EDTA (pH 5.2), and was extruded ten times through a 0.1 μm pore size polycarbonate membrane at 45°C to form small unilamellar liposomes (SULs). The resultant liposome suspension was chromatographed on a Sepharose CL6B-packed column (1.6 × 30 cm) equilibrated with Hepes-buffered saline (HBS) pH 6.8. Liposomes eluting at void volume were collected, and were then incubated with 1 mg of DOX for 1 h at 45°C. The liposomes were separated from unencapsulated (free) DOX by Sepharose CL6B chromatography as described above, and were then incubated with 2 mg of thiolated IgG for 1 h at 37°C followed by an additional incubation with 5 μl of 2-mercaptoethanol for 30 min. Antibody-coated DOX-loaded liposomes (CILs) were purified by Sepharose CL6B chromatography with HBS pH 7.4, sterilised by filtration through a 0.2 μm pore size polycarbonate membrane, and stored at 4°C until use. The lipid, antibody and DOX contents of liposomes were determined as described previously (Hashimoto *et al.*, 1983; Tanaka *et al.*, 1989). CILs contained 108–136 μg of antibody and 25–32 μg of DOX per μmol of total lipid.

*Quantitative analysis for CIL association*

Adherent cells were detached with 0.004% actinase, 0.1% EDTA in PBS for 5 min at 37°C, and were washed once with ice-cold PBS. Cells were mixed with CILs in a volume of 0.2 ml in standard medium containing 10% FCS and incubated for 2 h at 4°C. After washing with ice-cold PBS twice, cells were mixed with 0.3 M hydrochloric acid, 50% ethanol to extract DOX, and then incubated for 20 min at 37°C. After centrifugation at 500 *g* for 10 min, the fluorescence intensity of DOX (and its metabolites) in the supernatant was determined fluorometrically at 480 nm (excitation) and 580 nm (emission). An external standard curve for DOX was drawn by plotting the percentage recoveries of DOX from control samples mixed with known doses of DOX.

*Flow cytometric analysis for cell-surface CILs*

Cells were treated with CILs in standard medium containing 10% FCS for 2 h at 4°C with vortexing at 15 min intervals. Cells were washed twice with ice-cold PBS, and reincubated for 0–2 h at 37°C in standard medium containing 10% FCS. After washing as above, cells were treated with FITC-AL-6 (50 μg ml$^{-1}$) for 1 h at 4°C. After washing twice, cell fluorescence was analysed by a FACScan flow cytometer (Becton Dickinson, Mountain View, CA, USA) with excitation at 488 nm and emission at 515–545 nm. The fluorescence intensity of 10 000 viable cells was recorded. All determinations were done at the same detection sensitivity. The mean fluorescence intensity of each sample was computed.

*Analysis for processing of CILs by cell*

Processing of CILs by cells was determined by flow cytometry on the basis of intracellular fluorescence of liposomal DOX as described below. As the fluorescence of DOX encapsulated in CILs is self-quenched at high concentrations, its dilution results in augmentation of the fluorescence signal (dequenching). Thus, when CILs are endocytosed by cells and are released into an acidic environment in endosomes or lysosomes, DOX (a lipophilic weak base) will leak from them, resulting in dilution and an increase of fluorescence inside cells.

Cells were treated with CILs for 2 h at 4°C, washed with ice-cold PBS, resuspended in standard medium containing 10% FCS and incubated for various times at 37°C. Cell fluorescence was then measured by flow cytometry as described above except for detection at FL2 range (emission at 545–590 nm).

*Analysis for cytotoxicity of CILs*

The cytotoxic activities of CILs were determined by assaying the inhibition of protein synthesis on cancer cells. [$^3$H] Leucine but not [$^3$H]thymidine incorporation highly correlated with the viable cell number after the treatments as estimated by trypan blue staining. This measurement therefore also includes cytostatic effects.

Reciprocal dilutions of free DOX solution, CILs or CL suspension (100 μl) and 1 × 10$^5$ cells suspended in 100 μl of standard medium containing 10% FCS were mixed in a test tube and incubated for 30 min at 37°C. The cells were washed twice with standard medium, centrifuged at 200 *g* for 5 min, and resuspended in 1 ml of standard medium containing 10% FCS. Aliquots of the cell suspension were distributed in quadruplicate into a Falcon flat-bottomed 96-well tissue culture plate (4 × 10$^3$ cells per well), and then cultured in 200 μl of fresh standard medium containing 10% FCS for 3 days in a humidified carbon dioxide incubator. After the culture period, supernatant was discarded, cells in each well were starved of leucine by incubation with leucine-free medium (100 μl) for 2 h, pulsed with [$^3$H]leucine (0.5 μCi per well) for an additional 4 h, and then harvested by actinase–EDTA treatment. The radioactivity of the cells was measured by standard liquid scintillation counting. In the case of Figure 3, the residual cells (840 μl of cell suspension) were also collected by centrifugation and fluorometrically analysed for DOX association.

*Analysis for PHA response of PBMCs*

The cytotoxic activities of drugs on normal PBMCs were determined by assaying the inhibition of their PHA response. PBMCs (2.0 × 10$^6$) were mixed with free DOX solution, CILs or CL suspension in 200 μl of standard medium for 30 min at 37°C. After washing twice with ice-cold standard medium, cells were seeded at 2.5 × 10$^5$ cells per well in quadruplicate into a Falcon flat-bottomed 96 well-tissue culture plate, and cultured in 200 μl of standard medium containing 10% FCS and PHA at 20 μg ml$^{-1}$. After 3 days' culture, cells were pulsed with [$^3$H]thymidine (0.5 μCi per well) for 4 h, and then harvested. The radioactivity of the cells was measured by standard liquid scintillation counting.

### Results

Binding of SER4 CILs (anti-gp185 CILs) was analysed on SKBr-3 and MKN-7 cells, which expressed both gp185 and gp125, T24 cells, which expressed only gp125 (almost gp185-negative tumour cells), and PBMCs (antigen-negative normal cells) and compared with HBJ127 CILs (anti-gp125 CILs), CLs or free DOX (Figure 1). Among the four cell types, SER4 CILs selectively bound to SKBr-3 and to MKN-7 at a level 2.8 times lower than to SKBr-3. HBJ127 CILs bound to all three cancer cells: T24, SKBr-3 and MKN-7. Both CILs showed far weaker binding to PBMCs, at almost the same level as that of CLs and free DOX, which showed background levels. The amount of bound SER4 CILs was 2.5 and 5.9 times less than the amount of HBJ127 CILs in SKBr-3 and MKN-7 respectively. As this binding analysis was done under saturating conditions (data not shown), the results represent the binding capacity of each CIL on the target cells.

In a cytotoxicity analysis, SER4 CILs inhibited protein synthesis of SKBr-3 in a dose-dependent manner with an $IC_{50}$

of 0.8 μg ml$^{-1}$ DOX (Figure 2a, open triangles). This cytotoxic activity was completely blocked by competition with excess intact SER4 MAb (Figure 2a, closed triangles), demonstrating the specificity of SER4 CILs. This specificity was also confirmed by the far lower $IC_{50}$ of SER4 CILs for T24 cells and PBMCs (18 and 12 μg ml$^{-1}$ respectively; Figure 2c and d). SER4 CILs show selective cytotoxicity also on MKN-7, but to a lesser degree than on SKBr-3 ($IC_{50}$, 4.8 μg ml$^{-1}$ in Figure 2b). The $IC_{50}$ values of free DOX among three cancer cells were almost the same (0.45–0.62 μg ml$^{-1}$ DOX) but slightly lower than that for PBMCs (1.3 μg ml$^{-1}$). CLs showed only weak cytotoxicity against all four target cells. Especially in the case of SKBr-3, HBJ127 CILs, which had shown higher binding capacity to SKBr-3 (Figure 1), showed relatively low cytotoxic potential ($IC_{50}$ 1.5 μg ml$^{-1}$; Figure 2b) compared with SER4 CILs. Thus, cell-associated DOX at each dose in Figure 2a and b was quantified to reveal the amount of DOX required for 50% inhibition of [$^3$H]leucine incorporation (Figure 3). In the case of SKBr-3 cells, it was $1 \times 10^8$ DOX molecules per cell for SER4 CILs, which was 4.5 times lower than that required for HBJ127 CILs (Figure 3a). Similar results were obtained for MKN-7 cells, the concentration required being 4.3 times lower for SER4 CILs than for HBJ127 CILs (Figure 3b).

We then demonstrated endocytosis of CILs by SKBr-3 and MKN-7 cells (Figure 4). When SKBr-3 cells were coated with CILs at 4°C and reincubated at 37°C (Figure 4a), both CILs on the cell surface were decreased over an increasing incubation time, but the initial rate of decrease was higher for SER4 CILs (64%/10 min) than for HBJ127 CILs (30%/10 min). Also, on MKN-7 cells, the amount of SER4 CILs bound on the cell surface decreased faster than HBJ127 CILs, but slightly less efficiently than on SKBr-3 cells (Figure 4b). Under the same conditions as Figure 4, decrease of cellular DOX content was within 5% of the initial value in both cell lines (data not shown). Thus, such decreases suggest the internalisation of CILs by endocytosis as this process was inhibited with ammonium chloride, chloroquine and colchicine (data not shown) (Berinstein *et al.*, 1987; Collins *et al.*, 1989).

We further analysed processing of both CILs using flow cytometric techniques. As shown in Figure 5a, dequenching of DOX fluorescence on SKBr-3 cells was observed in SER4 CILs by about 270%, but was not found for HBJ127 CILs. Under fluorescence microscopy, at time 0 in Figure 5a, DOX fluorescence of SER4 CILs was observed as ring shapes,

**Figure 1** Binding of CILs to target cells. Cells ($2 \times 10^5$ for SKBr-3, MKN-7 and T24, $2 \times 10^6$ for PBMCs) were mixed with free DOX ( ■ ), SER4 CILs, ( □ ), HBJ127 CILs ( ▨ ) or CLs ( ▧ ) at a final DOX concentration of 30 μg ml$^{-1}$. After 2 h incubation at 4°C, cell-associated DOX was quantified as described in Materials and methods. Columns and bars are the mean and s.e., respectively, from three determinations.

**Figure 2** Effect of CILs on protein synthesis of tumour cells and proliferation of PBMCs with PHA. Cells treated with free DOX, CLs or CILs in various DOX concentrations were analysed for their [$^3$H]leucine (**a**, **b** and **c**) or [$^3$H]thymidine (**d**) incorporation activity as described in Materials and methods. The percentage of mean incorporation of radioisotope as compared with non-treated cells (control) is shown. Symbols represent the average values from four determinations. All s.e. values were less than 8.8% of control. (**a**) SKBr-3. (**b**) MKN-7. (**c**) T24. (**d**) PBMCs. ●, Free DOX; ○, CLs; □, HBJ127 CILs; △, SER4 CILs; ▲, SER4 CILs with excess (1 mg ml$^{-1}$) SER4 MAbs.

**Figure 3** Inhibition of protein synthesis by SER4 CILs (△) and HBJ127 CILs (□) on SKBr-3 (**a**) and MKN-7 (**b**) cells depending on DOX association. During the analysis described in Figure 2 the amount of DOX associated with cells was concomitantly analysed using residual cell suspensions. Plots for cell-associated DOX vs per cent [$^3$H]leucine incorporation are shown.

**Figure 4** Down-modulation of cell-surface CILs at increasing incubation times. Intact (open symbols) or formalin-fixed (closed symbols) cells were incubated with CILs (0.2 mM lipid) for 2 h at 4°C, washed twice with ice-cold PBS, and reincubated in standard medium containing 10% FCS for the indicated period at 37°C. Cells were then treated with FITC-AL-6 and processed for flow cytometry as described in Materials and methods. Per cent mean fluorescence intensities as compared with the values at time 0 are shown. Symbols and bars represent the mean values and s.e. of the mean, respectively, from three determinations. (**a**) SKBr-3. (**b**) MKN-7. □, ■, HBJ127 CILs; △, ▲, SER4 CILs.

indicating localisation on the cell surface. At 1 h it was found to be brighter and was observed in small dots beneath the cell membrane or near the nucleus, probably indicating localisation in endosomes or lysosomes. On the other hand using HBJ127 CILs after 1 h incubation, the fluorescence was observed both on the cell surface and as intracellular dots with a similar intensity. Under the same conditions MKN-7 showed only weak processing of SER4-CIL (Figure 5b).

### Discussion

We first examined the potential of anti-gp185 CILs (SER4 CILs) for therapeutic application. Since gp185 is also expressed on normal cells, although at a far lower level, the magnitude of the therapeutic efficacy is based on the degree of overexpression of gp185 on the target cancer cells. As shown in Figure 1, SER4 CILs showed higher binding to SKBr-3 and MKN-7 which overexpressed gp185, than to the control cancer cell T24 and normal PBMCs. SER4 CILs also showed cytotoxicity to SKBr-3 and MKN-7 cells in an antigen-dependent manner, but showed only limited toxicity against T24 cells and normal PBMCs. Although the findings presented in this paper represent only the first step in further application of CILs, the selective cytotoxicity against SKBr-3 and MKN-7 in conjunction with results discussed below suggests the potential of SER4 CILs for therapeutic application. In particular, when CILs were injected i.v. into a mouse, the (liposomal) DOX clearance rate was 12 times longer than that of free DOX (our unpublished data), suggesting superior *in vivo* pharmacokinetics of CILs for cancer therapy as compared with free DOX.

We next examined the dependency of CIL endocytosis on CIL cytotoxicity by comparing the two CILs targeting different antigens, gp185 and gp125, on the same target cells. As shown in Figure 3a, the amount of SER4 CILs associated with SKBr-3 was smaller than HBJ127 CILs at all doses

**Figure 5** Analysis of processing of CILs by cells. Cells were incubated with CILs (0.2 mM lipid) for 2 h at 4°C, washed twice with ice-cold PBS, and reincubated in standard medium containing 10% FCS for the indicated period at 37°C. Then cells were washed twice with ice-cold PBS and processed for flow cytometry. Per cent mean fluorescence intensities as compared with the values at time 0 are shown. Symbols and bars represent the mean values and s.e. of the mean, respectively, from three determinations. (**a**) SKBr-3. (**b**) MKN-7. □, HBJ127 CILs; Δ, SER4 CILs.

tested, however SER4 CILs showed stronger cytotoxicity than HBJ127 CILs comparing the same cellular DOX level (see results section). When CIL endocytosis by SKBr-3 was examined, SER4 CILs adhering to the cell surface were 90% internalised after a 1 h incubation, and were found to be efficiently processed (Figures 4 and 5), while HBJ127 CILs were only 60% internalised and were far less processed. Thus, in the case shown here, endocytosis positively correlated with cytotoxic efficiency of CILs, although these results differ from a previous report (Suzuki *et al.*, 1994).

Processing of CILs might be one of the key factors for the expression of CIL cytotoxicity as it has been reported that the cytotoxicity of DOX is dependent on its reduction by cytosolic enzymes (Bartoszek and Wolf, 1992). Thus, DOX in SER4 CILs endocytosed by target cells was found to have leaked out into endosomes, and then probably diffused into the cytosol resulting in more effective activation than for HBJ127 CILs. It is still unclear, however, what is the reason for the different intracellular fate of CILs between gp185 and gp125 as target antigens.

The percentage (90%) internalisation of SER4 CILs is high compared with that of anti-gp185 MAbs (Drebin *et al.*, 1985; Maier *et al.*, 1991; our unpublished data), of which only 14–70% were internalised. Thus, targeting in liposomal form might accelerate the internalisation of the ligand, perhaps because of multivalent binding (a CIL vesicle contained 30 IgG molecules) or their large size or cell membrane–lipid interactions.

Taken together, the cytotoxic efficiency of CILs was found to be dependent not only on antigen density, but also on the cell type studied and the antigen characteristics, in particular endocytosis. These considerations are thus important in selecting useful antigens (ligand) for therapeutic application of CILs.

In conclusion, SER4 CILs showed a selective anti-cancer effect against gp185-overexpressing SKBr-3 and MKN-7 cells, and, compared with gp125, gp185 was a more sensitive antigen for CIL cytotoxicity probably as a result of endocytosis activity.

**Abbreviations:** CIL, chemoimmunoliposome, doxorubicin-encapsulated immunoliposome; CL, chemoliposome, unmodified liposome containing doxorubicin; DOX, doxorubicin; PBMC, peripheral blood mononuclear cell; Hepes, *N*-2-hydroxyethylpiperazine-*N*'-2-ethansulphonic acid; MAb, monoclonal antibody; SPDP, *N*-hydroxysuccinimidyl-3-(2-pyridyldithio)propionate; FITC, fluorescein isothiocyanate; PBS, phosphate-buffered saline; HBS, Hepes-buffered saline (20 mM Hepes, 150 mM sodium chloride); MBS, *m*-maleimidobenzoyl-*N*-hydroxysuccinimido ester; MBPE, MBS derivative of dipalmitoylphosphatidylethanolamine; PHA, phytohaemagglutinin.

### References

BARTOSZEK A AND WOLF CR. (1992). Enhancement of doxorubicin toxicity following activation by NADPH cytochrome P450 reductase. *Biochem. Pharmacol.*, **43**, 1449–1457.

BERINSTEIN N, MATTHAY KK, PAPAHADJOPOULOS D, LEVY R AND SIKIC BI. (1987). Antibody-directed targeting of liposomes to human cell lines: role of binding and internalization on growth inhibition. *Cancer Res.*, **47**, 5954–5959.

CARLSSON J, DREVIN H AND AXEN R. (1978). Protein thiolation and reversible protein–protein conjugation. *N*-succinimidyl 3-(2-pyridyldithio)propionate, a new heterobifunctional reagent. *Biochem. J.*, **173**, 723–737.

COLLINS D, MAXFIELD F AND HUANG L. (1989). Immunoliposomes with different acid sensitivities as probes for the cellular endocytic pathway. *Biochim. Biophys. Acta*, **987**, 47–55.

DI FIORRE PP, PIERCE JH, KRAUS MH, SEGATTO O, KING CR AND AARONSON SA. (1987). A human growth-receptor like gene (*erb*B-2) is a potent oncogene when overexpressed in NIH/3T3 cells. *Science*, **237**, 178–182.

DI FIORRE PP, SEGATTO O AND AARONSON SA. (1991). Cloning, expression and biological effects of the erbB-2/neu gene in mammalian cells. *Methods Enzymol.*, **198**, 272–277.

DREBIN JA, LINK VC, STERN DF, WEINBERG RA AND GREEN MI. (1985). Down modulation of an oncogene protein product and reversion of transformed phenotype by monoclonal antibodies. *Cell*, **41**, 695–706.

HASHIMOTO Y, SUGAWARA M AND ENDOH H. (1983). Coating of liposomes with subunits of monoclonal IgM antibody and targeting of the liposomes. *J. Immunol. Methods*, **62**, 155–162.

MAIER LA, XU FJ, HESTER S, BOYER CM, MCKENZIE S, BRUSKIN AM, ARGON Y AND BAST JR RC. (1991). Requirements for the internalization of a murine monoclonal antibody directed against the HER2/neu gene product c-*erb*B-2. *Cancer Res.*, **51**, 5361–5369.

MASUKO T, ABE J, YAGITA H AND HASHIMOTO Y. (1985). Human bladder cancer cell-surface antigens recognized by murine monoclonal antibodies raised against T24 bladder cancer cells. *Jpn J. Cancer Res.*, **76**, 386–394.

MASUKO T, SUGAHARA K, KOZONO M, OTSUKI T, AKIYAMA T, YAMAMOTO T, TOYOSHIMA K AND HASHIMOTO Y. (1989). A murine monoclonal antibody that recognizes an extracellular domain of the human c-*erb*B-2 protooncogene product. *Jpn J. Cancer Res.*, **80**, 10–14.

SUZUKI S, MASUKO T, TAKANASHI K, TAKASHIO K AND HASHIMOTO Y. (1992). Assay of cell surface-bound immunoliposomes using monoclonal antibody reacts with a cross-linking reagent. *Chem. Pharm. Bull.*, **40**, 1893–1896.

SUZUKI S, WATANABE S, UNO S, TANAKA M, MASUKO T AND HASHIMOTO Y. (1994). Endocytosis does not necessarily augment the cytotoxicity of adriamycin encapsulated in immunoliposomes. *Biochim. Biophys. Acta*, **1224**, 445–453.

TANAKA T, SUZUKI S, MASUKO T AND HASHIMOTO Y. (1989). In vitro targeting and cytotoxicity of adriamycin in liposomes bearing monoclonal antibody against rat or human gp125 cell proliferation-associated antigen. *Jpn J. Cancer Res.*, **80**, 380–386.